# Institutional Housekeeping

**Published:** 1911-11-18

**Authors:** 


### INSTITUTIONAL HOUSEKEEPING.

(Contributions invited: Workers' Questions will be welcome.)

### How to Avoid Wasting Bread.

There are many ways in which bread can be wasted in all institutions. Waste to a certain extent cannot be avoided, and it requires careful supervision to make use of pieces that might otherwise be thrown away and to prevent a great accumulation of them.

### In the Kitchen.

Bread, where economy is studied, should not be eaten before it is thirty-six hours old, and it becomes stale and unpalatable after three days. In most institutions bread baked on Wednesday is used on Friday, and a successive supply of loaves thus arranged for, Sunday's supply coming in on Friday night. Large loaves are more advantageous than small ones, and those of a long shape can be cut up with less waste and less crust. The bread should be stored in a cool place out of the sun on glazed brick tiles if possible.

The amount of bread required for household or institution use should be carefully estimated when numbers fluctuate, to prevent stale loaves being left over or the supply of fresh bread being encroached upon. Where the contract is a large one the baker will generally agree to exchange stale loaves for fresh ones daily. In the larder should stand a special covered receptacle for pieces of bread, crusts, and scraps. A large earthenware pan, a wooden butter tub or box, or a large flour tub can be utilised. This must be kept clean, emptied every day or every two days, wiped out carefully and loose crumbs removed, and scalded or scrubbed once a week, for unless this is done the bread quickly becomes mouldy and unfit for use. All crusts, heels of loaves, trimmings from toast, etc., that cannot be made use of at the moment must be placed in this receptacle and use made of them as soon as possible. Crumbs or tiny fragments should not be kept for fear of turning mouldy.

There are a great many ways of using the accumulated pieces, principally in the form of puddings. When the housekeeper arranges the menu for the day, puddings in which bread can be introduced should be regularly served to some of the patients and household each day, though not necessarily in the same form. In every fixed menu provision should be made in it for a daily use of pieces of bread. It is difficult to detect the use of bread in many puddings if the fragments are carefully prepared beforehand. The bread pieces and crusts should be cut or broken up and soaked for half-an-hour or so before use in cold water; hot water makes the bread too soft and pasty, and the puddings consequently heavy. The bread must then be squeezed dry through a colander or sieve with a large wooden spoon or vegetable presser and should be well beaten with a fork until every lump is removed.

It can thus be introduced into plain custards, into bread puddings of various kinds, boiled, baked, or steamed, with the addition of figs, fresh or dried fruits, lemon, marmalade, or jam. It can take the place of breadcrumbs in most recipes if less of the liquid ingredients is used. Again, the bread may be moistened with boiling stock instead of water, beaten well, and used as a thickening for soups, when it should be boiled for a few minutes in the soup before serving.

Bread and milk can also be made from dry pieces, provided they are well soaked before use. Breadcrumbs can be made from dried scraps of bread, pounded in a mortar or beaten with a rolling-pin and passed through a wire sieve. These would be too dry for puddings, but are useful for hams and bacon, and for coating fish and other foods before frying.

In utilising cooked meat, bread moistened with stock or with milk, and well beaten, can be introduced with vegetable flavourings and a meat cake made which can be baked or steamed. Rissoles can also be made with it coated with egg and dried breadcrumbs and fried.

### In the Pantry.

The pieces of bread for table use must be cut into convenient pieces, neither too large or all may not be eaten, nor too small or they will become quickly dry and unappetising. It is the custom in some large schools and other institutions to cut crusts with about one inch of fresh bread and use these for dinner, so that the crumb may be used for cooking or for cutting bread and butter.

The bread must not be cut long before use, and in hot weather it should be covered, as if left exposed to the air for an hour it becomes dry and hard.

Pieces left over from the dinner table should never be placed in the larder piece-box, as the mere suspicion that this is done renders every dish containing bread highly unpopular. It is possible to carry economy too far when it defeats its own ends.

### In the Ward Kitchen.

There is a great opportunity for waste here, chiefly in the preparation of more than is wanted for the patients' use. Loaves are sometimes very wastefully handled so that a great deal of *débris* is left over. Whatever comes out of the wards must be thrown into the waste pail and so down to the pig-tub, but a receptacle should be provided for any portions of loaves left over in the ward kitchen that they may be kept and used for the next meal, or returned immediately to the kitchen. Constant supervision must be exercised by the ward sister to prevent waste and the surplus bread ought not to be the perquisite of the wardmaid, as that is a direct incentive to lavish handling of it. It is sad to calculate the number of pounds of bread daily thrown away in institutions or placed in that necessary, though baneful, adjunct to the kitchen, the pig-bucket, for want of forethought.

